# Influence of EGR3 Transfection on Imaging and Behavior in Rats and Therapeutic Effect of Risperidone in Schizophrenia Model

**DOI:** 10.3389/fpsyt.2020.00787

**Published:** 2020-09-24

**Authors:** Guangfei Li, Xiaowei Han, Wenwen Gao, Zeyu Song, Shuai Zhao, Feiyi Sun, Hong Ma, Ailing Cui, Xiaoying Tang, Guolin Ma

**Affiliations:** ^1^School of Life Science, Beijing Institute of Technology, Beijing, China; ^2^Department of Radiology, China-Japan Friendship Hospital, Beijing, China; ^3^Graduate School, Peking Union Medical College, Beijing, China; ^4^Changzhi Medical College, Changzhi, China; ^5^Anatomy Department, Changzhi Medical College, Changzhi, China

**Keywords:** schizophrenia, risperidone, resting state fMRI, regional homogeneity, functional connectivity

## Abstract

Schizophrenia is a type of neurodevelopmental psychiatric disorder. However, to date, scientists have not discovered the etiology and effective treatment of this condition. We injected the early growth response gene (EGR3) into the bilateral hippocampus to build a schizophrenia rat model. Behavioral phenotyping and resting-state functional magnetic resonance imaging (rs-fMRI) were used to analyze the behavioral and cerebral alterations in the schizophrenia rat model. The efficacy of risperidone therapy was also evaluated. We divided 34 rats into four groups: schizophrenia model group (E group), sham-operation group (FE group), healthy control group (H group), and risperidone therapy group (T group). Open field test and Morris water maze were conducted as behavioral experiments. Next, we performed rs-fMRI after four weeks of EGR3 transfection and risperidone treatment and analyzed imaging data using regional homogeneity (ReHo), the amplitude of low-frequency fluctuations (ALFF), and functional connectivity (FC). We examined the difference in behavioral and neural activation among the four groups and considered the correlations between behavior and imaging results. EGR3 gene transfection decreased the total moved distance in the open field test and the duration in the Q5 zone of the Morris water maze. Risperidone treatment reversed the trend and improved the performance of rats in these behavioral tests. Schizophrenia induced several neural alterations in ALFF and ReHo metrics of the rat brain, and risperidone could partly reverse these alterations. The results suggest that similar research is required for schizophrenia and that risperidone may be a novel treatment for dysregulated neural activation in schizophrenia.

## Introduction

Schizophrenia is a complex psychiatric disorder that affects approximately one percent of the world’s population. Genetic and environmental factors adversely affect neural development, and they are thought to contribute to the pathogenesis of schizophrenia (1). The disease is characterized by ‘social brain’ disorder and disturbances in functional connectivity (FC) (2).

Animal models, especially those using rats, can help researchers study the features of psychiatric diseases and develop novel effective therapies. The early growth response gene (EGR3) is not only crucial for synaptic plasticity but also very important in neuronal activation and brain development (3). Previously, it has been found that the EGR3 gene was abnormally expressed in the brain of schizophrenic patients (4). In a study by Ma (5), schizophrenia rat models were developed by injecting lentivirus particles that carried the EGR3 gene into the hippocampus and dentate gyrus of the rat brains.

Resting-state functional magnetic resonance imaging (Rs-fMRI) has been widely used to study neurodegenerative diseases and neuropsychiatric disorders such as Alzheimer’s disease (AD) (6), mild cognitive impairment (MCI) (7), depression (8), schizophrenia (9), and medial temporal lobe epilepsy (10). The amplitude of the low-frequency fluctuations (ALFF) (11), and the regional homogeneity (ReHo), which were developed by Zang et al. (12, 13), are the two of the main metrics used for analyzing spontaneous brain activity (13, 14). Both these metrics have been used to analyze brain activity in participants with mental diseases (7, 8, 11, 15–17). To comprehensively assess pathophysiological brain dysfunction, ALFF and ReHo were used as complementary methods (18, 19). Apart from functional alteration, research has also shown that the brain structure of schizophrenia subjects changes: the volume of the bilateral hippocampus in schizophrenia patients was significantly smaller than that of health controls (20–23).

Previous reports have shown that risperidone has better clinical effects compared to traditional antipsychotics (20), providing the basis of the hypothesis that risperidone could relieve cognitive dysfunction symptoms in schizophrenia patients. Since ALFF and ReHo can be used to identify activated brain regions, we used the two metrics to compare neural activation among schizophrenic rats, sham-surgery rats, healthy rats, and risperidone-treated rats. We also used the ReHo value altered clusters as seeds to calculate the decrease in FC regions. We conducted the open field test and Morris water maze to evaluate the rats’ ability to explore an unfamiliar environment and their working memory capacity.

## Methods

### Animals

We conducted controlled and randomized animal experiments at the Beijing Institute of Technology, in accordance with the Guidance Suggestions for the Care and Use of Laboratory Animals, as issued by the Ministry of Science and Technology of the People’s Republic of China. Sprague Dawley rats (38 males, aged 4 weeks, weighing 100 ± 10 g) from Beijing Weitong Lihua Experimental Animal Technology Co., Ltd (experimental animal production license: SCXKBeijing 2016–0006) were purchased and bred in the experimental animal room. The animal habitat was maintained at a temperature of 20–24°C and 40–55% humidity. All efforts were made to minimize animal suffering, and decapitation was performed after the rats were anesthetized with isoflurane.

### EGR3 Transfection

We randomly divided the rats into four groups, and then injected lentivirus particles carrying the EGR3 gene into their bilateral hippocampus and dentate gyrus to build the schizophrenia model as described earlier (5). We also injected lentivirus particles carrying the green fluorescent protein (GFP) instead of EGR3 into the same brain regions to build the sham-operation group. As for the risperidone treatment group, the EGR3 transfected rats were administered an intraperitoneal injection of risperidone (0.2 mg/kg, Sigma, USA) for 14 consecutive days after a two-week recovery period. Dead subjects were removed after the above procedure, and the remaining 34 rats were divided into the schizophrenia model group (E, 10, rats + EGR3 gene + normal saline), sham-operation group (FE, 9, rats + GFP gene + normal saline), healthy control group (H, 6, rats without operation + normal saline), and risperidone treatment group (T, 9, rats + EGR3 + risperidone).

### Behavioral Tests

To confirm differences in brain function among the four groups, we tested the rats by making them go through the platform in the Morris water maze and crossing squares in the open field test. The behavioral tests were conducted on the day after the end of injections of normal saline or risperidone and before the rs-fMRI analyses. Each rat was put in the open field test device that comprised 25 grids, and the number of crossing grids (NCG) within 2 minutes was recorded to measure the ability to explore strange space. Each rat was trained four times a day with different entry points for finding the platform, and this protocol continued for three days. Then, the frequency (FQ5) and total time (TQ5) the rats spent in the Q5zone (the place hiding the platform) in one minute was recorded on the fourth day to measure the working memory.

### MRI Acquisition

We used a GE 3T scanner (GE, Discovery MR750, America) and a standard rat coil (Shanghai Chenguang Medical Science and Technology, Shanghai, China) to acquire the structural and functional images. Anesthesia was induced by intraperitoneal injection of 10% chloral hydrate (0.3 ml/100 g) before scanning. To reduce head movement, the rats were fixed with a vacuum pillow and band during their positioning in the scanner (head first, prone).

A Field-Map, one rs-fMRI time series, and a 3D structural image were scanned using the MRI acquisition protocol. We used the Spin Echo method to acquire the T2W1 coronal, axial, and sagittal images. The parameters for the T2W1 image were: repetition time (TR) = 3225 ms, echo time (TE) = 83 ms, field of view (FOV) = 80 × 80 mm^2^, number of slice = 32, slice thickness = 1 mm, interlayer space = 0 mm, number of signals averaged (NSA) = 1. Rs-fMRI image parameters: TR = 2000 ms, TE = 30 ms, FOV = 40 × 40 mm^2^, number of slices = 18, slice thickness = 1.5 mm, interlayer space = 0 mm, NSA = 1.

### Image Data Pre-Processing

Imaging data were pre-processed using Statistical Parametric Mapping (SPM8, Welcome Department of Imaging Neuroscience, University College London, U.K.) and the spmratIHEP toolbox, designed by Nie et al. (24). Images of each individual subject were first realigned (motion corrected). A mean functional image volume was constructed for each subject from the realigned image volumes. These mean images were co-registered with the T2W1 image and then segmented for normalization with affine registration, followed by nonlinear transformation. The normalization parameters determined for the structural volume were then applied to the corresponding functional image volumes for each subject. Finally, the images were smoothed with a Gaussian kernel of 3 mm at Full Width at Half Maximum.

Following current reporting standards, all imaging results were evaluated with voxel *p* < 0.001 (uncorrected), in combination with a cluster *p* < 0.05 [false discovery rate (FDR) corrected], based on Gaussian random field theory, as implemented in SPM (25).

### Behavior Test Data Analysis

We used SPSS 22.0 software to analyze the data. We used a one-way analysis of variance (ANOVA) to compare the four groups and check if data fitted normal distribution. To compare the two selected groups, we used an LSD post-hoc test to check the homogeneity of variance, and a Tamhane’s T2 test to check the heterogeneity of variance. Then, we used an independent-sample T-test to compare the differences between the two selected groups.

### fMRI Feature Calculation and Seed-Based Analyses

We calculated ALFF and ReHo, and then compared the differences between selected groups. We performed a seed-based analysis to assess local changes in brain connectivity. Firstly, since the ReHo values between matched groups had a statistically significant difference after FDR correction, we chose clusters of ReHo values as the regions of interest (ROIs) and used the mean ReHo value of ROI to analyze the correlation with the behavioral index. Next, we extracted the mean time courses for each rat and each of the ROIs and used them to analyze the seed-based FC. We calculated the FC of the whole brain based on ROIs and demonstrated FC changes in the four rat groups.

### Correlation Between Behavior and fMRI

We conducted a Pearson correlation analysis between behavioral task and ReHo values to examine the associations between behaviors and cerebral functional activations, which were caused by EGR3 gene transfection and risperidone therapy. We considered p < 0.05 as significant. The ReHo values used for analysis were the mean value of all the ROIs extracted from each subject’s brain images.

## Results

### Behavioral Tests Confirm Poor Working Memory and Decreased Capacity to Explore an Unfamiliar Environment

**Figure 1:**
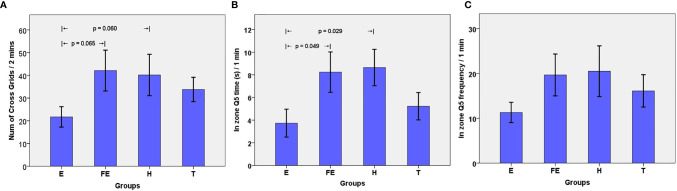
**(A)** Number of Grids crossed by rats in 2 minutes. Bar plots (mean) with error bars (standard error of the mean); **(B)** Time in Q5 zone by rats in 1 minute (unit: second); **(C)** Frequency in Q5 zone by rats in 1 minute.

We conducted an open field experiment to test the rats’ spontaneous activity. We chose NCG to understand the capability and adaptability of rats in exploring an unfamiliar environment. Results showed that the NCGs in group E rats were less than those in the FE group (p = 0.065) or H group (p = 0.060), showing a trend (**Figure 1**). This implied that the rats had a decreased ability to explore unacquainted environments. No significant differences among other groups were found (**Table 1**). The NGC’s of the T group rats were increased compared with those of group E, indicating that risperidone had a therapeutic effect in schizophrenic rats.

**Table 1:** Effects of EGR3 gene transfection and risperidone treatment on Behavioral test.

Group		Morris Water Maze		Open Field Test
		Q5 in zone frequency	Q5 in zone time	Num of grids
	ANOVA			
E vs. FE vs. H vs.T	F	1.166	2.604	1.912
	*p*	0.339	0.070	0.149
	T-test			
E vs. FE	t	-1.667	-2.116	-2.032
	*p*	0.114	**0.049**	0.065
E vs. H	t	-1.770	-2.430	-2.042
	*p*	0.098	**0.029**	0.060
E vs. T	t	-1.154	-0.862	-1.741
	*p*	0.264	0.401	0.100
T vs. H	t	-0.689	-1.730	-0.648
	*p*	0.503	0.107	0.528

Bold cluster p value meaning p < 0.05.

We also used the Morris water maze test to estimate working memory, which reflects individual cognitive function. The results showed that the FQ5 and TQ5 in group E were less than those in the other three groups (**Table 1**). Particularly, the TQ5 in group E was significantly shorter than those in group FE (p = 0.049) and group H (p =0.029) (**Figure 1**). As for FQ5, rats in group E performed less than those from other groups. However, this difference was not significant (**Figure 1**). The two indexes in group T were greater than those in group E, whereas they were less than those of group H. This suggested that although using risperidone treatment could partly reverse this phenomenon, it would be hard to achieve normal levels.

### Comparison of ALFF/REHO Reveals Changes in Prefrontal Function

Behavioral test results showed that risperidone administration at a dose of 0.2 mg/kg could partly improve behavioral changes caused by schizophrenia, but no statistically significant differences were observed. Taking this into account, we conducted rs-fMRI on rats in the four groups to explore the effect of ECG3 gene transfection and risperidone administration on the brain.

**Figure 2:**
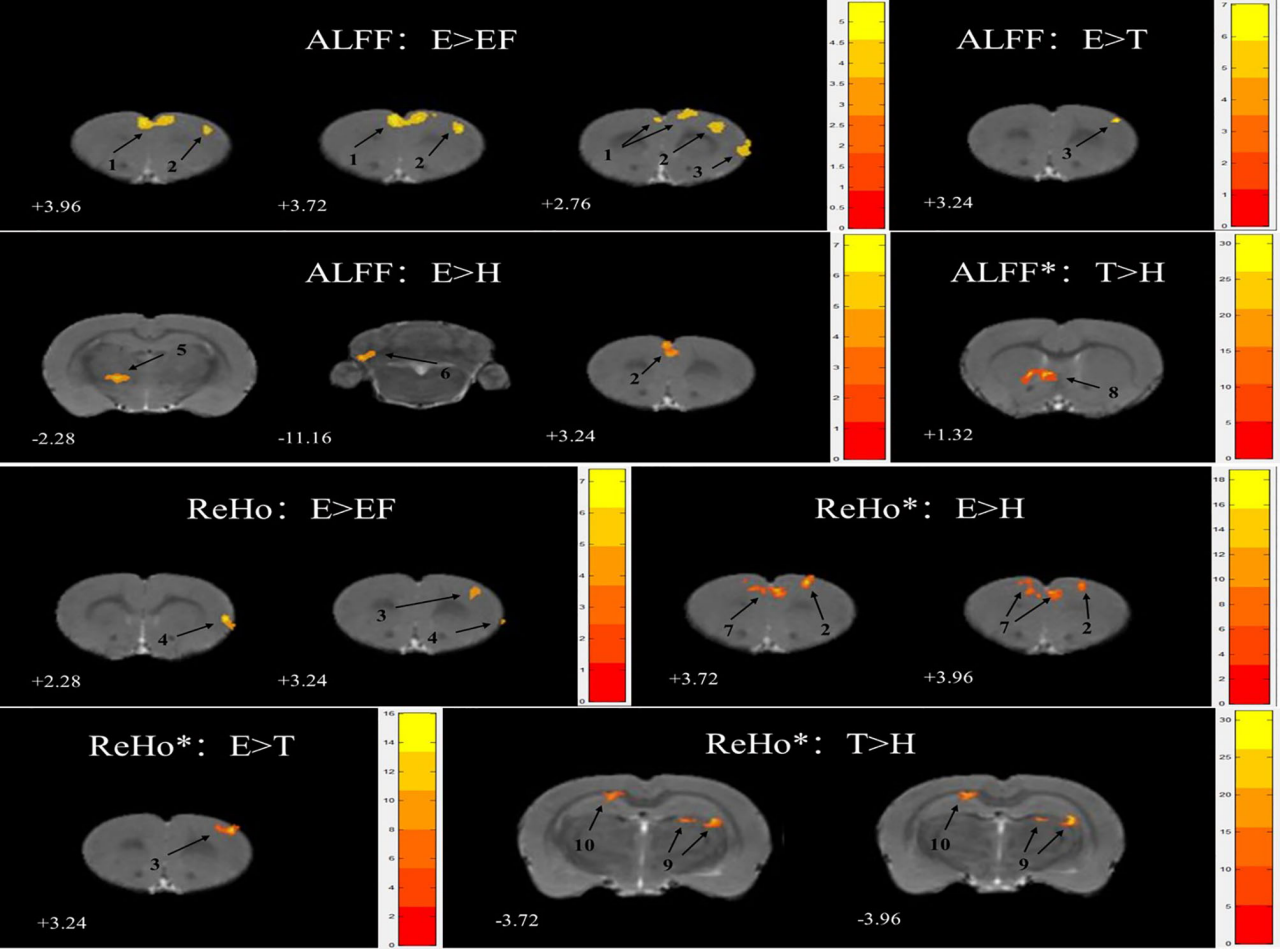
Statistical maps of voxel t-value of ALFF and ReHo comparisons of two chosen groups, * stand for the difference was significant after FDR correction. The numbers at the bottom left of each image refer to the z coordinates in the stereotaxic space of Paxinos and Watson (the 5^th^ edition). The color bars were used to signify the t-value of the group analysis (the color is brighter, the t-value is higher). The left side of the images corresponds to the left side of the brain, and vice versa. The number on each brain image stand for the different brain regions as follows: 1. frontal association cortex (FrA) 2. secondary motor cortex (M2) 3. primary motor cortex (M1) 4. primary somatosensory cortex, jaw region (S1J) 5. ventral anterior thalamic nucleus (VA) 6. preculminate fissure (pcuf) 7. prelimbic cortex (PrL) 8. caudate putamen striatum (CPu) 9. oriens layer of the hippocampus (Or) 10. cingulum (cg).

No significant activation in ALFF was seen between the first three matched groups: E vs. FE, E vs. H, and E vs. T. When compared with the H group, the T group showed a greater ALFF value in the caudate putamen striatum (CPu) after FDR correction (**Table 2**; **Figure 2**).

**Table 2:** Effects of EGR3 gene transfection and risperidone treatment on ALFF and ReHo.

Group		Cluster Name	Cluster size		ClusterP- value	Paxinos coordinate(mm)		
						X Y Z		
ANOVA				Peak F value				
E vs. FE vs. H vs. T	ALFF	S1Tr	151	9.83	0.486	4	1	-3
		M2	200	10.80	0.484	2	2	4
		6a	121	10.20	0.484	-1	3	-13
		M1	173	9.49	0.532	-3	2	3
	ReHo	FrA_(L)_	158	21.41	**0.022**	-2	3	5
		FrA_(R)_	131	18.30	0.078	2	3	5
		Crus2	194	22.65	**0.014**	-1	4	-13
T-test				Peak t value				
E vs. FE	ALFF	FrA	297	5.48	0.127	-1	2	4
		M2	193	5.06	0.359	3	3	4
		M1	157	4.99	0.536	5	4	3
	ReHo	S1J	110	7.40	0.873	5	4	2
		M1	58	4.54	0.873	3	2	3
E vs. H	ALFF	VA	156	7.36	0.202	-2	6	-2
		pcuf	287	6.07	0.091	-4	5	-11
		M2	260	5.70	0.106	-1	1	3
	ReHo	M2	179	18.82	**0.024**	2	2	4
		PrL	355	17.83	**0.001**	-1	3	4
E vs. T	ALFF	M1	53	7.04	0.918	4	2	3
	ReHo	M1	234	16.04	**0.024**	4	2	3
T vs. H	ALFF	CPu	459	31.27	**1.058e-4**	-2	4	1
	ReHo	Or	205	22.40	**2.967e-5**	4	3	-4
		cg	162	17.04	**1.439e-4**	-2	2	-4

Bold cluster p value meaning p < 0.05.

There were several cerebral regions activated in ReHo values when an ANOVA analysis was taken between E, FE, H, and T groups (**Table 2**). Some cerebral region alterations exhibited a similar range for ALFF and ReHo, such as the matched group E versus FE in the primary motor cortex (M1), matched group E versus H in the secondary motor cortex (M2), matched group E versus T in M1. Except for E versus FE, the other three matched groups for ReHo measures showed a significant difference after FDR correction. ReHo value in group E increased in the M1 and primary somatosensory cortex and the jaw region (S1J) compared with group FE. There was also an increase in the M2 and prelimbic cortex (PrL) compared with group H, and in the M1 compared with group T (**Figure 2**). The group T showed an increased ReHo value in the oriens layer of the hippocampus (Or) and cingulum (cg) compared with group H (**Figure 2**).

### Correlation Analyses Demonstrate the Link Between Behavior and fMRI

**Figure 3:**
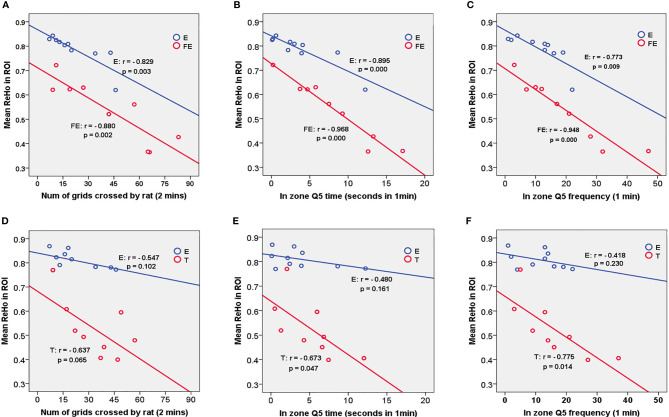
Correlations between ReHo mean value in ROIs of E vs. FE (S1J, M1), E vs.T (M1) and behavioral indexes. Significant results were presented in this figure about matched groups E vs. FE. **(A)** demonstrates a negative correlation between mean ReHo value and number of grids crossed by rat within two minutes in open field test. The same trend was found in **(B, C)** in Morris water maze. A negative correlated trend was found in group T between mean ReHo value in M1 and number of grids **(D)**, besides, significantly negative correlation between mean ReHo value and time duration at zone Q5 in panel **(E)** and through frequency at zone Q5 in panel **(F)** were presented in group T.

Since the ReHo values in the three matched groups showed a significant difference, the mean ReHo value of the altered brain regions was used to analyze the correlation with behavioral test indexes. In matched group E versus FE, the mean ReHo value of the S1J and M1 clusters was calculated to analyze the Pearson linear correlations with the behavioral indices of Morris water maze and open field test. In group E versus T, the mean ReHo value of M1 was extracted to analyze the correlation with behavioral indices (**Figure 3**).

For group E in matched group E versus FE, the significant correlations were shown between the FQ5, TQ5, NCG and the mean ReHo value in the S1J and M1 (FQ5: r = -0.773, p = 0.009; TQ5: r = -0.895, p = 0.000; NCG: r = -0.829, p = 0.003). For group FE in matched group E versus FE, significant correlations were shown between the FQ5, TQ5, NCG and the mean ReHo value in the S1J and M1 (FQ5: r = -0.948, p = 0.000; TQ5; r = -0.968, p = 0.000; NCG: r = -0.880, p = 0.002).

For group T in matched group E versus T, significant correlations were shown between the FQ5, TQ5 and the mean ReHo value in the M1 (FQ5: r = -0.775, p = 0.014; TQ5: r = -0.673, p = 0.047), (**Figure 3**).

The mean ReHo values of ROI seed regions did not show a significant difference from behavioral test indexes in other matched groups (**Table 3**).

**Table 3:** Correlations between ReHo mean value in ROIs and behavioral outcomes.

ROI	group		ReHo mean vs. Q5 in zone frequency	ReHo mean vs. Q5 in zone time	ReHo mean vs. Num of squares
S1J/M1	E	r	-0.773**	-0.895**	-0.829**
		*p*	**0.009**	**0.000**	**0.003**
	FE	r	-0.948**	-0.968**	-0.880**
		*p*	**0.000**	**0.000**	**0.002**
M2/PrL	E	r	-0.281	-0.264	-0.315
		*p*	0.431	0.465	0.376
	H	r	-0.736	-0.731	-0.530
		*p*	0.077	0.099	0.280
M1	E	r	-0.418	-0.480	-0.547
		*p*	0.230	0.161	0.102
	T	r	-0.775*	-0.673*	-0.637
		*p*	**0.014**	**0.047**	0.065
Or/cg	T	r	-0.619	-0.639	-0.006
		*p*	0.075	0.064	0.987
	H	r	-0.396	-0.634	-0.510
		*p*	0.437	0.176	0.302

**p < 0.01, *p < 0.05. ROIs were clusters of increased ReHo value in each matched group.

Bold cluster p value meaning p < 0.05.

### Seed-Based Analyses Show Decreased Functional Connectivity

The ReHo value of brain clusters that showed differences between matched groups in **Table 2** were chosen as ROI seeds. All the four matched groups showed a lower FC (for corresponding brain regions) with ROI seeds, without significant differences after FDR correction

**Figure 4:**
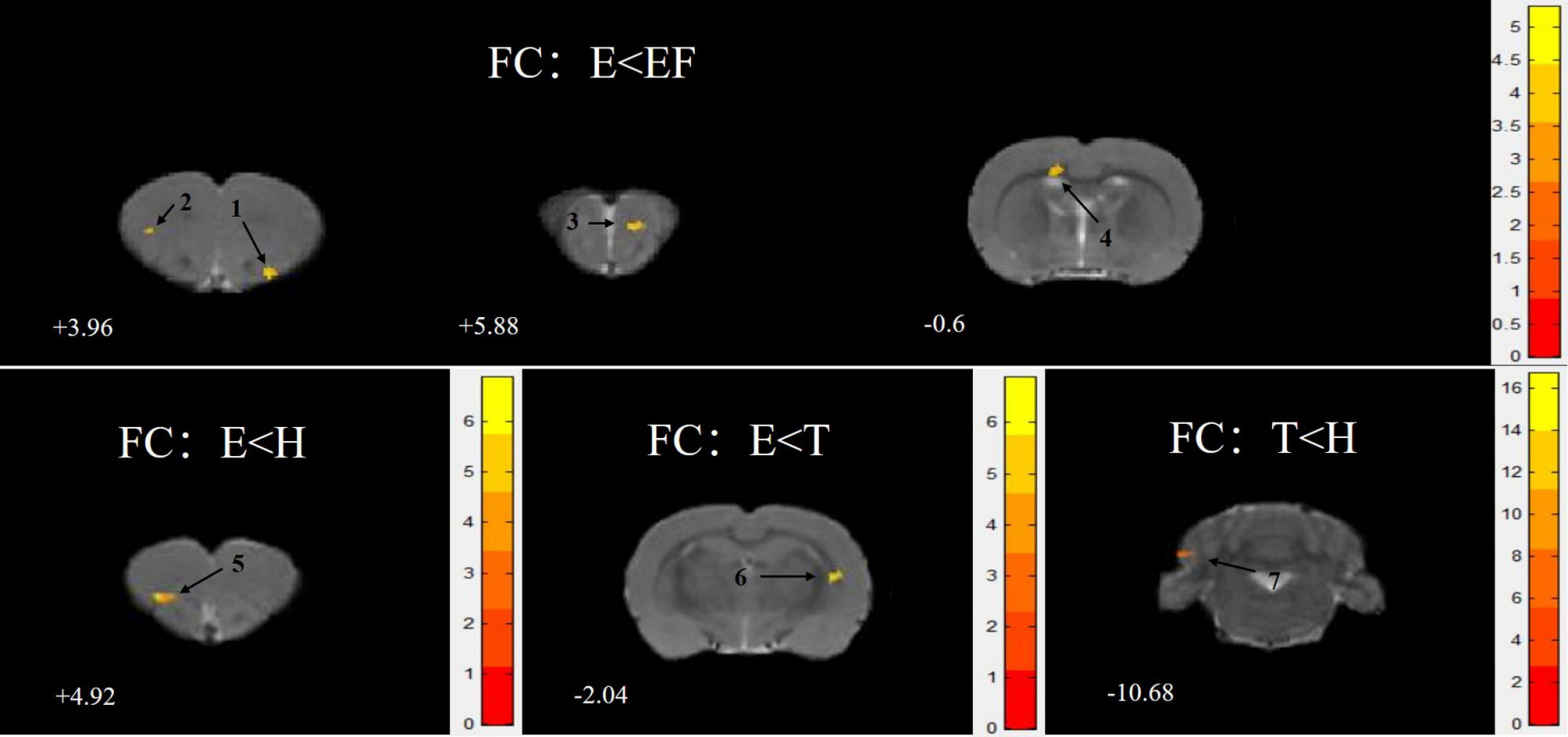
Statistical maps of voxel t-value of FC comparisons of two chosen groups, all the clusters were greater than 50 and showed a decrease trend level. The numbers at the bottom left of each image refer to the z coordinates in the stereotaxic space of Paxinos and Watson (the 5th edition). The color bars were used to signify the t-value of the group analysis (the color is brighter, the t-value is higher). The left side of the images corresponds to the left side of the brain, and vice versa. The number on each brain image stand for the different brain regions as follows: 1. anterior olfactory nucleus, dorsal part (AOD) 2. secondary motor cortex (M2) 3. granular insular cortex (GI) 4. primary somatosensory cortex, hindlimb region (S1H1) 5. dorsolateral orbital cortex (DLO) 6. caudate putamen striatum (CPu) 7. Crus 1 of the ansiform lobule (Crus1).

For rats in group E compared with group FE, the ROI seeds (S1J, M1) showed lower FC in the anterior olfactory nucleus, dorsal part (AOD), M2, granular insular cortex (GI), primary somatosensory cortex, and hindlimb region (S1HL). For rats in group E compared with group H, ROI seeds (M2, PrL) showed lower FC in the dorsolateral orbital cortex (DLO) (**Figure 4**). For rats in group E compared with group T, the ROI seeds (M1) showed lower FC in the CPu (**Table 4**). For rats in group T compared with group H, the ROI seed (Or, M1) showed lower FC in the crus 1 of the ansiform lobule (Crus1).

**Table 4:** Functional connectivity decline regions in each matched group.

Group	(seed name)FC regions	Cluster size	T value	P value	Paxinos coordinate(mm)		
					X	Y	Z
E vs. FE	(S1J/M1)						
	AOD	51	4.94	0.219	2	5	4
	M2	61	5.33	0.181	-4	2	4
	GI	57	4.66	0.195	1	2	6
	S1HL	107	4.56	0.083	-2	1	-1
E vs. H	(M2/PrL)						
	DLO	115	6.89	0.054	-3	4	5
E vs. T	(M1)						
	CPu	72	6.91	0.572	4	4	-2
T vs. H	(Or/cg)						
	Crus1	360	16.77	0.081	-5	4	-11

## Discussion

We observed that ReHo values increased in the prefrontal regions, including the frontal association cortex, motor cortex, and somatosensory cortex in the E group rats. This also confirmed that the prefrontal lobe plays an important role in schizophrenia, and increased activation of the prefrontal cortex might be associated with the pathogenesis of schizophrenia. We also found that ReHo values increased in some regions of the limbic system, including the hippocampus in T group rats. The hippocampus has a close relationship with information storage (26). We found a significant negative correlation between mean ReHo value and behavioral indexes in both the E and FE groups; the increased ReHo in the frontal region could be a biomarker for cognitive impairment. Functional alteration of the motor cortex, linked with depression (18, 27) and generalized anxiety disorder, showed a decreasing ReHo value in the motor cortex (28). In contrast, in our study, the increased ReHo value in the motor cortex may be a neuroimaging indicator of decreased volitional activity, which is the main symptom in schizophrenia. The ALFF activation was increased in the prefrontal lobe and thalamus in the E group. After therapy with risperidone, the activation was suppressed, whereas the activation of the striatum in the treated T group was still greater than that in the healthy H group. The ReHo value of the prefrontal cortex was increased in the E group. After risperidone therapy, the phenomenon was suppressed, whereas the ReHo value of the hippocampus in the T group was greater than that in the H group. The difference in ALFF and ReHo among groups may suggest that the thalamus and prefrontal lobe, which are responsible for cognitive and decision-making functions recover first. Although risperidone can reverse some brain functional impairment, the hippocampus is responsible for memory processing storage, and processing of spatial information, and may need a much longer time for recovery, or in the case of severe injury to the hippocampus, the reversal may be impossible. According to behavioral test results, symptoms that are linked with memory and attention disorders in the risperidone treatment group were also restored to a certain extent. Therefore, the increased activation in the prefrontal lobe cortex and hippocampus might be an indicator that can be used for schizophrenia diagnosis (1).

We determined the lower seed-based FC in the olfactory bulb (AOD and GI) and frontal brain areas (M2, S1H1, and DLO) of schizophrenia model rats. Alterations of the frontal and olfactory bulb FC based on prefrontal brain seed areas (M1, S1J, and PrL) induced by EGR3 transfection may explain how the underlying neural mechanism of schizophrenia could affect brain development and function, as the frontal cortex is important in the major circuits involved in cognition (29). The decreased frontal connectivity seen in our results is consistent with neurodevelopmental animal models of schizophrenia (30–32), indicating that prefrontal cortex disruptions may be a core mechanism of schizophrenia (33, 34). An earlier study (30) showed similar imaging activations in a neurodevelopmental schizophrenia model and proposed that these could be a biomarker for preclinical models. Corroborating this hypothesis, our results showed the same FC alterations in EGR3 transfection rats. Another review study (35) showed that schizophrenia subjects presented with disruption in the central brain regions (36–43) and alterations in the temporal and parietal hubs (36–39, 43). Therefore, our future studies will focus on the regional network organization in schizophrenia model rats.

Our study has a few limitations. Firstly, we only used the enhanced ReHo regions as seeds to analyze the decreased FC of model rats. Since activity did not occur in isolated areas of the brain, it would be better to consider regional and whole-brain networks in the future. Secondly, we only used risperidone as a treatment for the schizophrenia model due to the limited quantity of rats. Using other groups to explore whether risperidone could alter cognitive function and behavior without EGR3 gene transfection would provide more rigorous evidence.

In summary, this study conducted the open field test and Morris water maze experiments to evaluate the ability of rat subjects to explore an unfamiliar environment and their working memory capacity. The TQ5 in the E group was shorter than that in the FE and H groups. The TQ5 and FQ5 in group T were greater than those in group E, whereas they were less than those in group H. This implied that risperidone treatment could partly reverse this phenomenon, but it was difficult to revert to normal levels. We used the ALFF and ReHo method to demonstrate the activation of several brain regions in the schizophrenia rat model. The limbic system and prefrontal cortex, which contribute to the development of symptoms of schizophrenia were found to be activated. Our study revealed that the ALFF and ReHo activations in the cerebral regions may be potential biomarkers for cognitive impairment. Therefore, future research in imaging related to the pathology of schizophrenia is warranted. We also demonstrated that risperidone could be a complementary treatment for schizophrenia patients. In addition, the decline of frontal and olfactory bulb FC based on prefrontal brain seed areas supports earlier research that implicated neural circuits including those in the prefrontal regions, as important pathways for mental disorders.

Despite the aforementioned limitations, the current study, with the help of EGR3 transfection and administration of risperidone, has demonstrated that several cerebral regions are involved in the pathogenesis of schizophrenia. In our opinion, the findings of this study not only have instructive value for imaging and pathological study of schizophrenia, but it can also provide important insights into the therapeutic effects of risperidone on cognitive function in schizophrenia patients.

## Data Availability Statement

The datasets analyzed in this manuscript are not publicly available. Requests to access the datasets should be directed to maguolin1007@qq.com.

## Ethics Statement

The animal study was reviewed and approved by Institutional Ethics Review Committee of the China-Japan Friendship Hospital.

## Author Contributions

GM, XT conceived the experiments. GM, HM, AC, and FS designed the experiments. GL, XH, WG, and SZ performed the experiments. GL, ZS analyzed the data. GL discussed the data. GL wrote the manuscript. All authors contributed to the article and approved the submitted version.

## Funding

This study was supported by the National Key Research and Development Program of China (Nos. 2020YFC2003903, 2019YFC0120903, and 2016YFC1307001), and the grants from National Natural Science Foundation of China (NSFC) (No. 81971585, 81571641, 81471743).

## Conflict of Interest

The authors declare that the research was conducted in the absence of any commercial or financial relationships that could be construed as a potential conflict of interest.
