# Supplementary figures and images for: Influence of EGR3 Transfection on Imaging and Behavior in Rats and Therapeutic Effect of Risperidone in Schizophrenia Model
Source: Front Psychiatry. 2020 Sep 24;11:00787. doi: 10.3389/fpsyt.2020.00787 (PMC7542223; doi:10.3389/fpsyt.2020.00787)

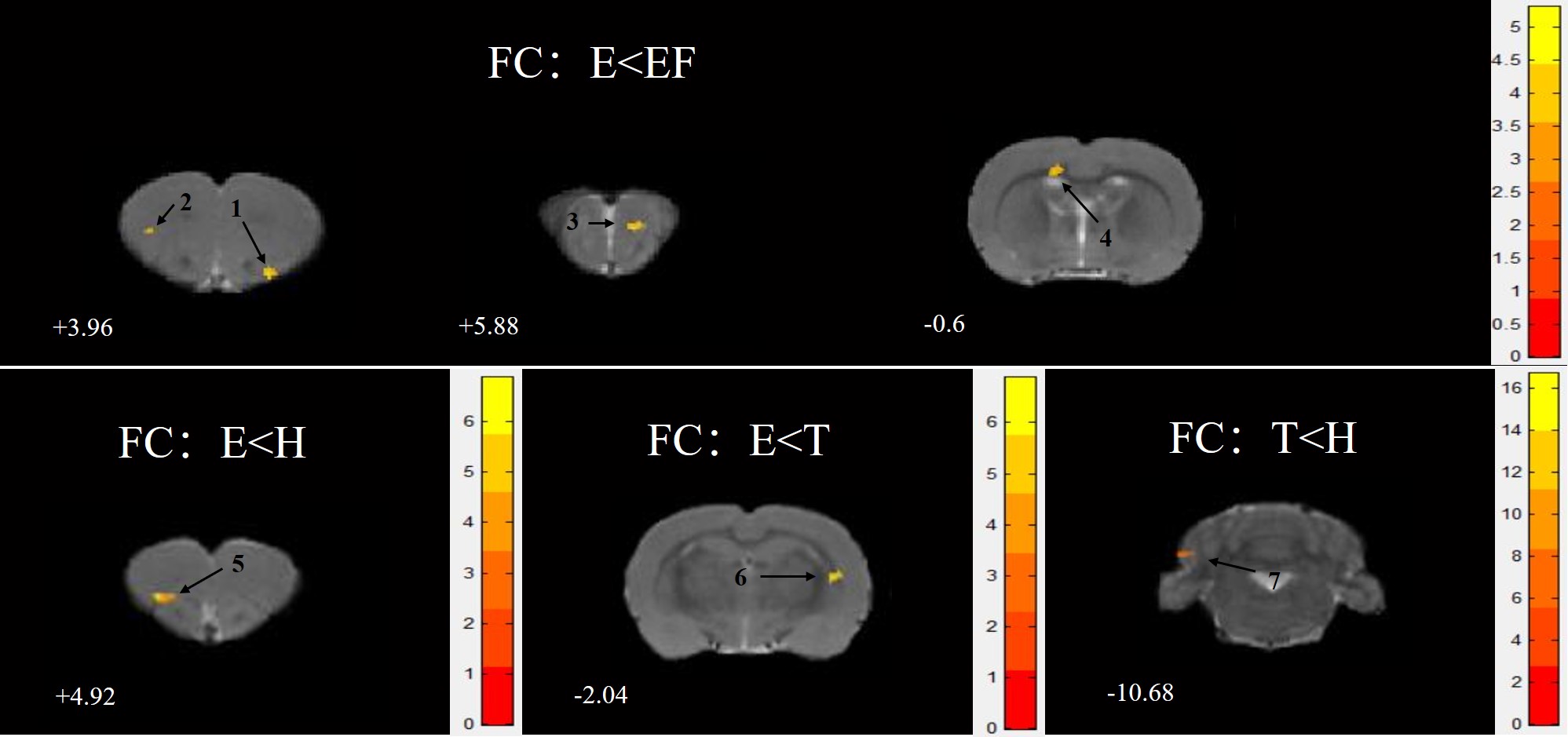

Supplement: Supplementary file 1 [file Figure_1.jpeg]

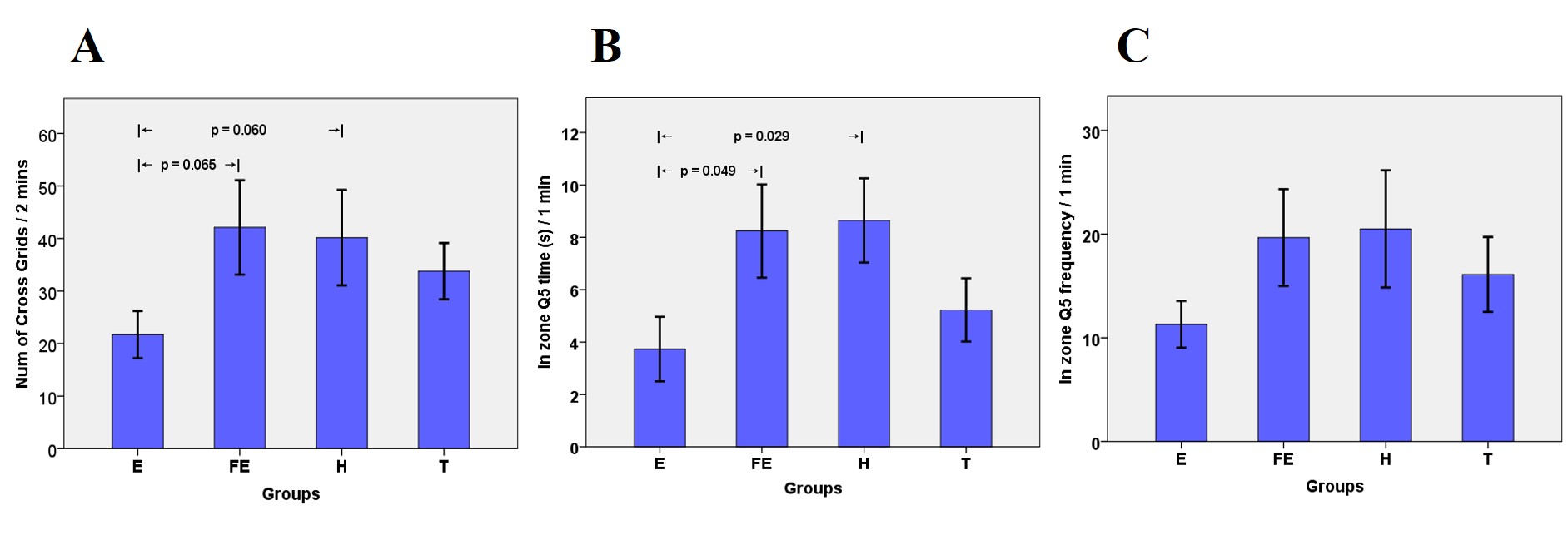

Supplement: Supplementary file 2 [file Figure_2.jpeg]

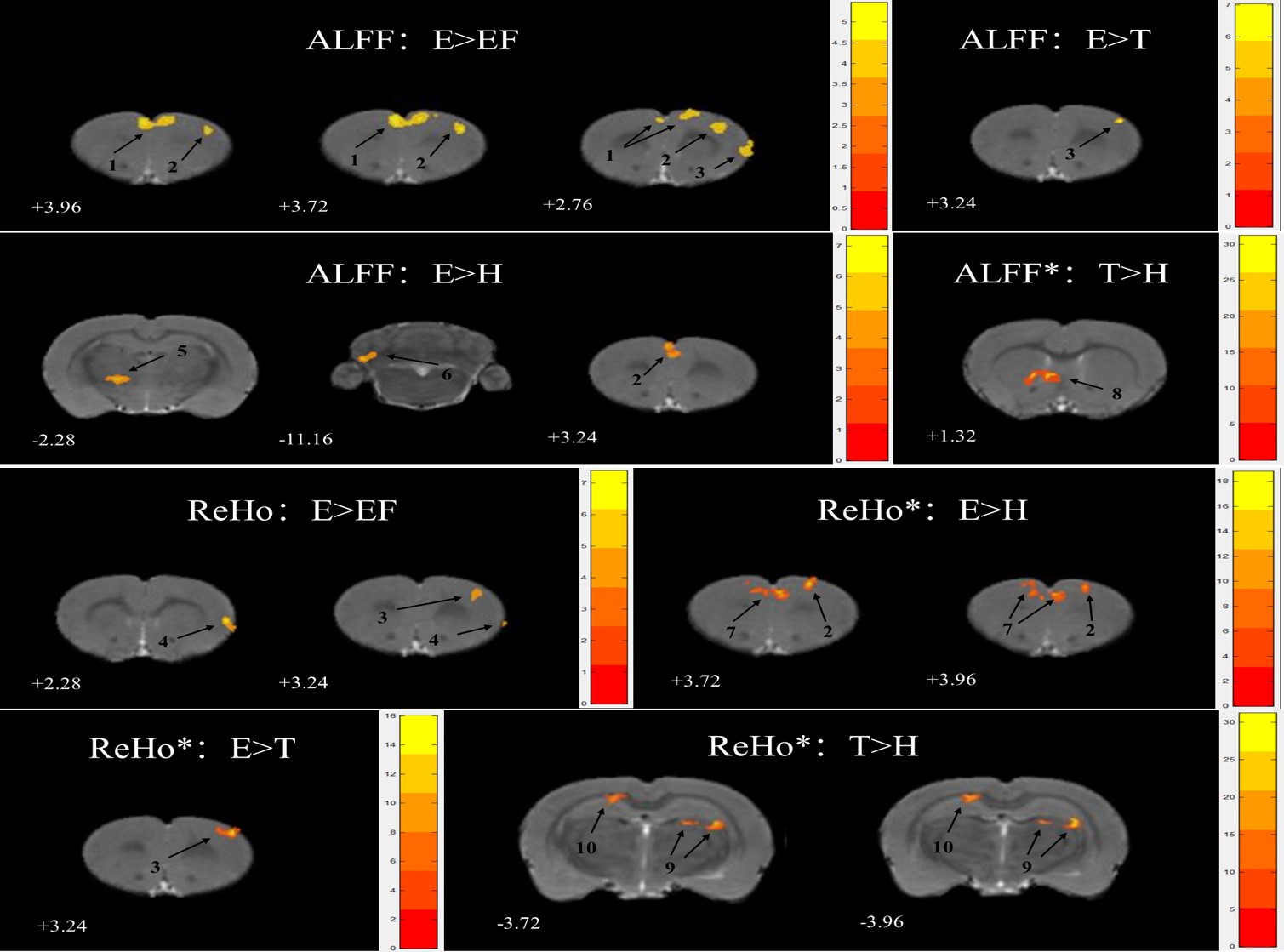

Supplement: Supplementary file 3 [file Figure_3.jpeg]

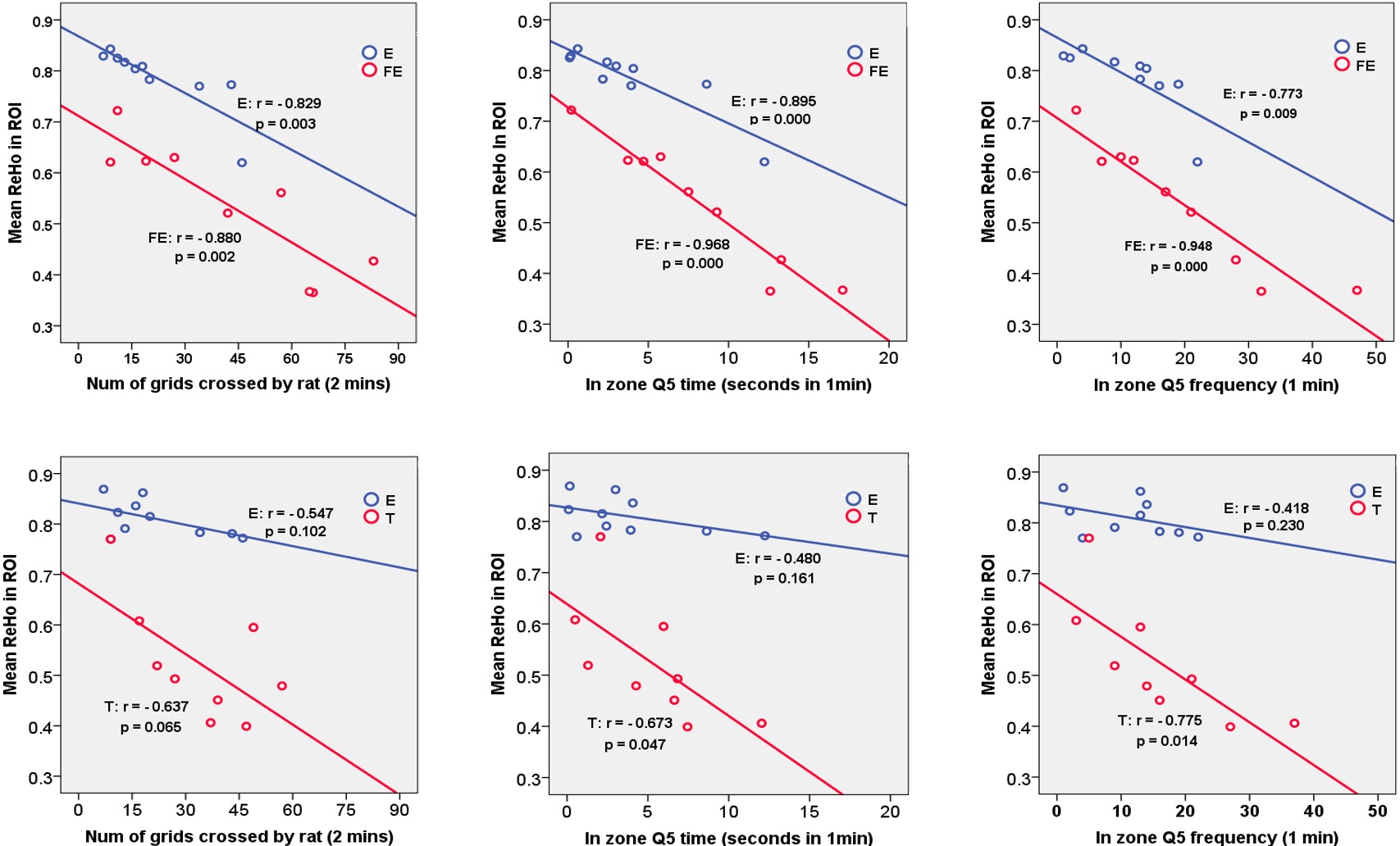

Supplement: Supplementary file 4 [file Figure_4.jpeg]
